# Optimized Sonochemical Exfoliation of Bulk 6H-SiC for the Synthesis of Multi-Layered SiC Nanosheets

**DOI:** 10.3390/nano15191480

**Published:** 2025-09-27

**Authors:** Eric Fernando Vázquez-Vázquez, Yazmín Mariela Hernández-Rodríguez, Omar Solorza-Feria, Oscar Eduardo Cigarroa-Mayorga

**Affiliations:** 1 Department of Nanoscience and Nanotechnology, CINVESTAV-Instituto Politécnico Nacional, Av. Instituto Politécnico Nacional 2508, Mexico City 07360, Mexico; fernando.vazquezv@cinvestav.mx; 2 Advanced Technologies Department, UPIITA-Instituto Politécnico Nacional, Av. Instituto Politécnico Nacional 2580, Col. Ticomán, Mexico City 07340, Mexico; yazmin.hernandez@cinvestav.mx; 3 Department of Chemistry, CINVESTAV-Instituto Politécnico Nacional, Av. Instituto Politécnico Nacional 2508, Mexico City 07360, Mexico

**Keywords:** SiC nanosheets, sonochemical exfoliation, multi-layered SiC nanosheets

## Abstract

In this study, a novel and rapid top-down synthesis method for the successful synthesis of few-layered 2D SiC is reported. Since the theoretical prediction of planar and stable 2D SiC with a direct bandgap, only a few experimental methods have overcome the challenging covalent sp^3^ hybridization of its bulk structure, unlike Van der Waals layered material bonding, making the synthesis of few-layered or mono-layered SiC more difficult due to the highly time- and energy-consuming methods. Moreover, correctly choosing between the more than 250 SiC polytypes increases the complexity of successful approaches to its 2D synthesis. This work reports, for the first time, multi-layered 2D SiC obtained using the wet ultrasonic probe sonochemical exfoliation method, reducing both the experimental synthesis time and energy consumption. Raman spectra showed the size-dependent correlation of the longitudinal optical (LO) mode, and IR showed the bond modification between bulk and nanostructured SiC. These results demonstrate a scalable and facile route for 2D SiC production; therefore, a wide variety of applications can be explored experimentally rather than theoretically, and methods such as the deposition of ScAlN layers onto SiN can be simplified in further studies.

## 1. Introduction

**Figure 1:**
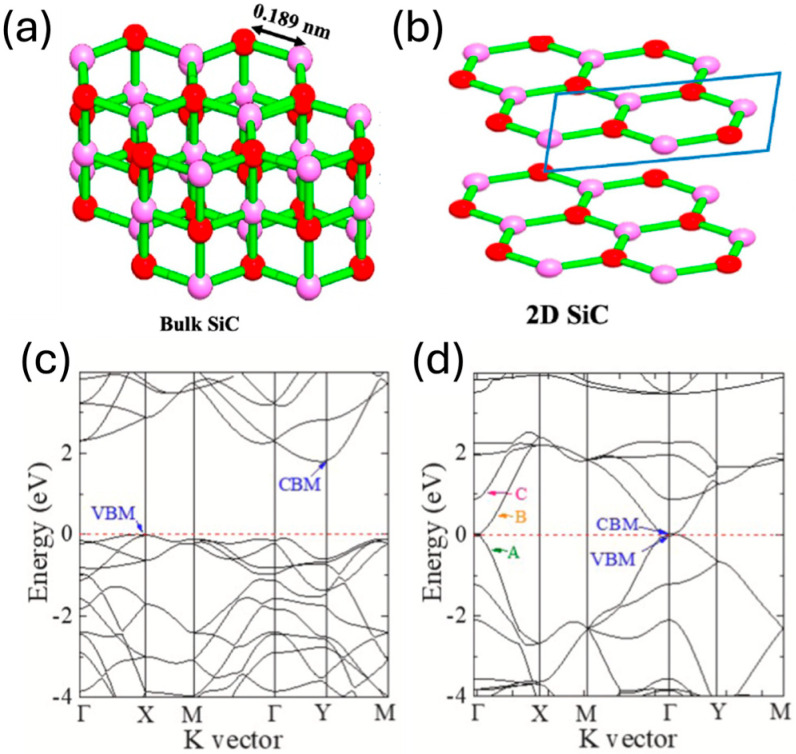
(**a**) Crystal cell structures of bulk 6H–SiC and (**b**) 2D SiC (republished from [21]) and electronic band structures of (**c**) bulk 6H–SiC and (**d**) 2D SiC that demonstrate the change from an indirect to direct bandgap (reprinted with permission from [32]; copyright 2021 Elsevier B.V).

Two-dimensional (2D) materials have been demonstrated to overcome the properties of their bulk counterparts. These advantages can be enhanced due to the atomically thin structure that allows more and stronger interactions, a larger surface area, tunable bandgaps, high conductivity, and fast charge separation, as seen in studies on heterosystems such as MoS_2_/WS_2_ heterostructures [1]. In this regard, 2D silicon carbide (SiC), a newly promising material for several applications in optoelectronics [2,3,4], microelectronics [4,5], and energy storage and production [6,7,8,9,10,11,12,13,14], has been widely studied, although experimental work remains barely explored [15,16]. Bulk SiC is a wide-bandgap semiconductor with high thermal conductivity, a large breakdown electric field, and a high saturation electron velocity, making it suitable for high-power [17,18,19], -frequency, and -temperature applications [17,18,20]. Two-dimensional materials drastically change the properties of materials in their bulk forms [21], making them more interesting for novel applications. In this way, 2D SiC has attracted attention, although its bulk structure with strong covalent sp^3^ hybridization and its indirect bandgap make experimental studies challenging [19,20,22]. Theoretical studies of 2D SiC have demonstrated that it exhibits a stable planar structure and has a direct bandgap, unlike its bulk counterpart with an indirect bandgap (Figure 1a–c) [21,22,23,24]. The primary challenge to 2D SiC’s synthesis stems from its bulk structure. In the last decade, several studies have successfully synthesized 2D SiC structures using different bottom-up and top-down synthesis methods. Bottom-up methods, such as chemical vapor deposition (CVD), have been widely used to successfully prepare 2D materials such as graphene [25], borophene [26,27], and selinene [28,29], among others, due to their selective growth mechanisms. Hence, thin layers of SiC have been obtained using the CVD method via the deposition of silicon (Si) and carbon (C) atoms onto a substrate under controlled conditions, such as the atmosphere and flow of the reactant precursor [28]. Moreover, one-step growth of 2D SiC via heteroepitaxy on a silicon substrate using CVD has been successfully reported [30]. Another bottom-up approach is molecular beam epitaxy (MBE), which represents a more controlled synthesis route for the growth of cubic 2D SiC (3C 2D SiC) on a silicon substrate via the thermal decomposition of SiC [31]. Despite these methods’ success, scalable approaches need to be explored for this material’s mass research. Top-down synthesis methods have shown promising routes for this. Wet exfoliation of bulk hexagonal SiC (6H–SiC) remains barely explored, and only a few approaches have been carried out. In these experiments, 6H–SiC is exfoliated with isopropanol or 1-methyl-2-pirrylidone (NMP) due to its ability to disperse and stabilize the materials. The synthesis time is approximately 24 h, resulting in a highly time- and energy-consuming method that remains deoptimized. Further analysis is required.

Two-dimensional SiC’s properties can be tuned by varying the stoichiometry and bonding [15]; for instance, multi-layer 2D SiC has an indirect bandgap in its bulk form and a direct bandgap in its monolayer form [4]. SiC nanostructures composed of tetragonal and hexagonal rings with C–C and Si–C bonds present strong ductility and anisotropicity and exhibit thermodynamic and mechanical stability at a low formation energy, which can enhance their scalable fabrication [32].

The application of 2D SiC represents an opportunity area, since materials such as gapless graphene, silicon, and bulk SiC are not fully applicable due to their drawbacks [15]. Moreover, the potential applications of 2D SiC in energy storage and conversion are outstanding due to its structure and previously superior properties [16]. Furthermore, SiC composite materials can enhance hydrogen and oxygen evolution reactions [33], resulting in platforms to explore a wide variety of catalysts.

To overcome the drawbacks and boost applications, this work explored the optimization of multi-layered 2D SiC via the wet sonochemical exfoliation of 6H–SiC. This study was undertaken to explore the synthesis time compared with other works in which single- and multi-layered 2D SiC powders were obtained via bath sonication. Using a vertical ultrasonic probe considerably reduced the reaction time by up to 2 h, effectively controlling the size and morphology of the obtained nanosheets.

## 2. Experimental Details

### 2.1. Materials and Reagents

Silicon carbide (SiC, ~400-mesh particle size, ≥97.5%, SIGMA-ALDRICH, St. Louis, MO, USA, SKU: 357391) was used as the precursor, and 1-methyl-2-pyrrolidone (NMP, ≥99.0%, SIGMA-ALDRICH, USA, SKU: 443778) as the stabilizer solvent, for the etching process to synthesize 2D SiC via the sonochemical exfoliation method. 2-propanol ((CH_3_)_2_CHOH, ≥99.5%, SIGMA-ALDRICH, USA, SKU: 278475) and deionized water (with a resistivity of 18 MΩ, SIGMA-ALDRICH, USA, SKU: W4502) were used for the cleaning process. All chemical reagents were used as received, without any additional purification steps.

### 2.2. Synthesis of SiC via US Probe Sonication

Two-dimensional SiC was synthesized via the following route: A solution was prepared by adding the 6H-SiC powder to NMP, achieving an average concentration of 1 mg/mL. The vertical probe sonicator (Cole-Palmer Ultrasonic Processor, 500 W, 20 KHz) was placed into the beaker with the previously prepared solution and activated with a square-wave mode with 4 s pulses (4 s on, 4 s off) at 100% amplitude for 2 h while maintaining a temperature of ~10 °C. Afterward, unexfoliated SiC was removed via centrifugation (Velocity 18R, Dynamica, fixed-angle FA18A rotor, 27.07× *g* max RCF) at a speed of 2000 rpm for 10 min at 10 °C. The further cleaning cycles to remove NPM were as follows: The solution was centrifuged at 13,000 rpm for 30 min at 10 °C, and the supernatant was removed; then, the pellet was washed with water or 2-propanol via bath sonication (VWR Aquasonic 50T Ultrasonic Cleaner, 90 W, 35 KHz) for 5 min at ~10 °C. This procedure was repeated until the solution had a pH of 7. Finally, the 2D SiC was transferred to a furnace for drying at 60 °C overnight.

### 2.3. Physical Characterization

The structural characterization, such as the crystal phase and structures of the as-synthesized nanoparticles, was conducted using a Panalytical X’pert X-ray diffractometer (Cu-kα; λ = 1.5418 Å; 2θ range step of 0.01°). For the morphological characterization, particle size determination, and elemental microanalysis, high-resolution images of the surface topography were obtained with a field-emission scanning electron microscope (SEM, JEOL JSM-6390L) using the secondary electron emission mode equipped with an energy-dispersive X-ray spectroscope (EDX, Bruker 5010 XFlash detector). Images were obtained at a 2 kV acceleration voltage at an 8 mm working distance. Furthermore, high-resolution imaging was conducted with a scanning transmission electron microscope (STEM, JEM-ARM200F) in the high-resolution mode to analyze the crystal structure and determine the crystallographic orientation of 2D SiC. Fourier-transform infrared (FT-IR GX, Perkin Elmer) spectra provided information regarding the chemical composition and structure of the bulk precursor and the obtained 2D material to understand the structural transformations after the ultrasonic etching. Raman spectroscopy (NT-MDT, NTEGRA spectra) provided the vibrational modes and chemical bonding, as well as structural information, to study the vibrational properties of the 2D synthesized structures. Finally, atomic force microscopy (AFM, NT-MDT Solver NEXT) was used to determine the thickness and lateral size of the exfoliated flakes to closely study the morphological structure. The analysis of the data obtained using the characterization techniques provided a detailed understanding of the structural and morphological changes produced by the synthesis technique until the formation of 2D structures. For the SEM, EDS, STEM, and AFM characterization tests, a solution of 2D SiC dispersed in 2-propanol with a concentration of 1mg/mL was prepared, and drops were deposited onto substrates, such as a silicon wafer and a holey carbon grid. For the XRD, FTIR, and Raman characterizations, dried 2D SiC silicon powders were directly used without further preparation techniques.

## 3. Results

### 3.1. SiC Synthesis

**Figure 2:**
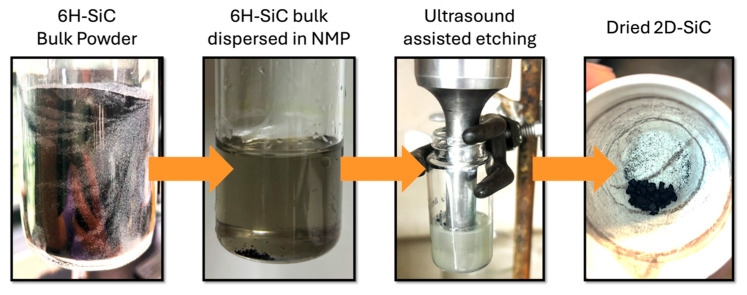
Step-by-step photographs of the ultrasonic-assisted wet exfoliation of bulk 6H–SiC powder using the Cole-Palmer vertical probe sonicator.

The step-by-step process for the synthesis of 2D SiC is shown in Figure 2. First, a bulk 6H-SiC precursor dispersion in NMP solvent was prepared via bath sonication for 10 min to obtain a well-dispersed solution, although a precipitate was seen at first glance. Then, the ultrasonic vertical probe was submerged to a depth of 1 mm into the SiC dispersion. After the completion of the ultrasonic-assisted wet exfoliation (US-w exfoliation), the semi-transparent solution changed to a cloudy solution, which, in experiments of this matter, can indicate successful exfoliation and the presence of nanoparticles. The cleaned and dried 2D SiC was then transferred onto a Si wafer for further characterization techniques.

**Figure 3:**
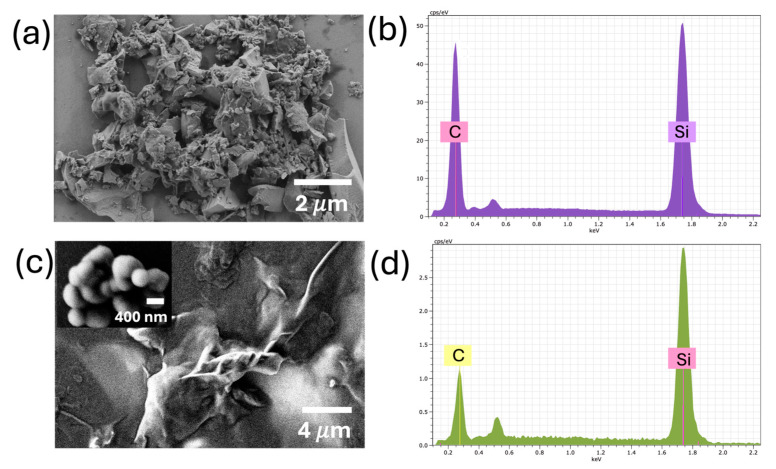
SEM micrographs (**a**,**b**) and EDX spectra (**c**,**d**) of bulk SiC and exfoliated SiC, respectively. Morphological changes can be seen from microscopic particles in bulk SiC to well-defined nanoparticles in as-synthesized SiC.

Figure 3 shows the results from the SEM and EDS characterization, in which a noticeable morphological change was observed. The 6H-SiC precursor consisted of non-homogenously shaped microstructures, with an average size of around ~2 μm (Figure 3a). The EDX spectrum (Figure 3b) shows that these microparticles consisted of Si and C, with an atomic ratio of 1:1, which demonstrated the high SiC purity. Meanwhile, the shape transformation was achieved after the US-w exfoliation. The sample surface showed a species of thin film on the Si substrate at a low magnification (Figure 3c), demonstrating a drastic morphological change. The inset in Figure 3c shows an isolated nanoparticle of ~400 nm, and the EDX spectrum (Figure 3d) reveals Si and C in an atomic ratio of 1:1, confirming highly pure SiC nanostructures. The oxygen content of the sample slightly increased, attributable to the amount of oxidation of the SiC.

### 3.2. Exfoliation Mechanism

**Figure 4:**
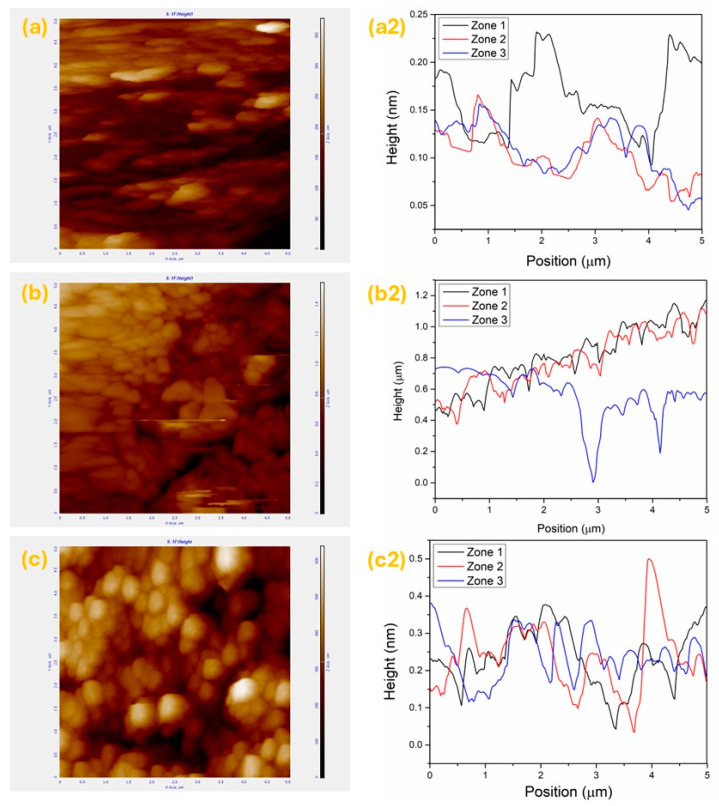
AFM images. Characterization of samples showing topographic (**a**–**c**) and cross-sectional profile (**a2**–**c2**) images (height profile of 3 random sections of the sample). Micrographs (**a**,**a2**) correspond to sample 1, (**b**,**b2**) to sample 2, and (**c**,**c2**) to sample 3.

AFM micrographs of SiC after 30 min, 1 h, and 2 h were obtained to investigate the exfoliation mechanism involved in US-w exfoliation (Figure 4). In Figure 4a, the AFM analysis of the supernatant after 30 min of US-w exfoliation shows large, irregular particles with a dispersed morphology, exhibiting lateral sizes close to ~1 μm and average heights of around ~250 nm. These results, when compared with the SEM image of bulk 6H–SiC (Figure 3a), confirm that only minimal morphological modification occurred. The relatively low energy transferred to the bulk particles during this stage was insufficient to induce significant structural changes, particularly the transition from sp^3^; to sp^2^; bonding [34]. After 1 h of US-w exfoliation, the AFM micrograph (Figure 4b) reveals a reduction in particle size and the appearance of more uniform morphologies. The lateral dimensions of the particles decreased to ~350–450 nm, while the mean height drastically dropped from ~250 nm to ~15 nm, as confirmed by the line profiles. This significant reduction demonstrates the onset of effective exfoliation, with the transferred energy being sufficient to disrupt the bulk structure and favor the thinning of the particles. In addition, the 2 h US-w exfoliated sample (Figure 4c) exhibited well-defined 2D-like SiC sheets with a relatively homogeneous circular morphology, decorated at the edges with nanoparticles of ~10 nm. The lateral sizes of the obtained sheets were ~400 nm, while the average thickness measured from the height profiles was ~5.5 nm, confirming the successful route toward 2D SiC synthesis via an optimized exfoliation time and precursor dispersion. Based on the interlayer spacing of bulk SiC (0.25–0.37 nm) [22,34], the obtained thickness corresponded to approximately 15–22 stacked layers. Although monolayers were not achieved, since extended exfoliation times tended to yield thicker aggregates (up to ~10 nm) that behaved as quasi-1D materials, these results demonstrate that tuning the exfoliation parameters offers a viable pathway to synthesize ultrathin 2D SiC nanosheets. The 3D view of the AFM images is presented in the Supporting Materials of the corresponding samples (Figures S1–S3 for samples obtained after 30 min, 1 h, and 2 h of US-w exfoliation).

### 3.3. Stabilization of 2D SiC Nanosheets

**Figure 5:**
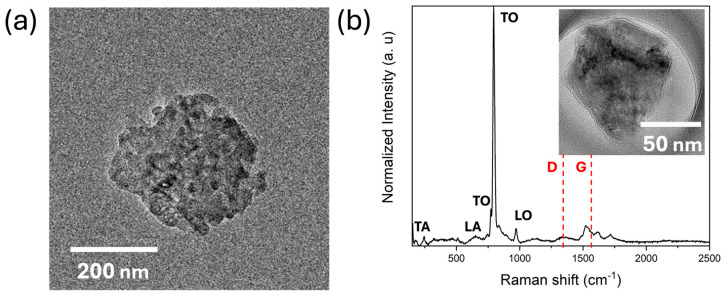
Characterization of 2D SiC using (**a**) TEM shows multi-layered structure and successful reduction in particle size; (**b**) Raman spectra highlight longitudinal acoustic (LA) and longitudinal and transverse optical (LO and TO) modes of the SiC and D and G bands of graphene.

Figure 5a is a low-resolution TEM image of various 2D SiC sheets with a circular-shaped morphology on the Si wafer substrate. The layers can differ as they are shifted parallel due to the stacking of the 6H–SiC with a sequence of ABCACB. Although not directly observed using TEM, the insert in Figure 5a may indicate the non-planar structure of the 2D SiC’s surface due to the stacking mechanisms [35]. The morphology and size observed by AFM were, thus, further confirmed using this technique. Meanwhile, the Raman spectrum of the 2D SiC in the 200–2500 cm^−1^ range is presented in Figure 5b. The peaks observed at 770, 780, and 970 cm^−1^ are typical of 6H-SiC. The 770 cm^−1^ and 780 cm^−1^ peaks are due to the Si–C bond in the transverse optical (TO) mode, and the 780 cm^−1^ peak is characteristic of all SiC materials. The 970 cm^−1^ peak belongs to the longitudinal optical (LO) of the A1 phonon in SiC, and the 250 and 600 cm^−1^ peaks belong to the transversal and longitudinal acoustic modes, TA and LA, respectively. Thus, this spectrum confirms the SiC nature of the exfoliated nanosheets; moreover, the widening of the peaks may be due to the quantum confinement effect in these nanostructures. Further analysis of this spectrum reveals that the weakening in the 970 cm^−1^ peaks can be attributed to the conversion from sp^3^ to sp^2^ hybridization, as mentioned in Chabi et al.’s work [3,22,36].

**Figure 6:**
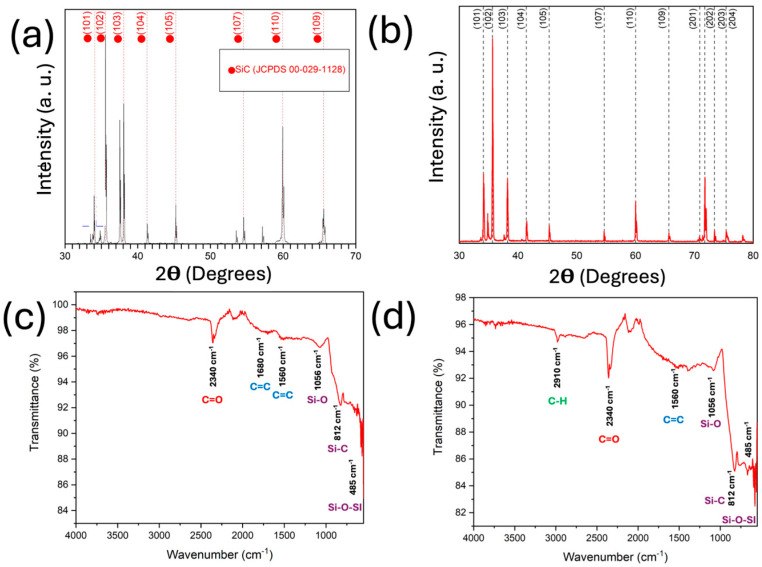
XRD patterns of (**a**) bulk and (**b**) 2D SiC show the main phase as 6H–SiC in both, although some peaks of other SiC phases appear via exfoliation. FTIR spectra of (**c**) bulk and (**d**) 2D SiC show increases in Si–O, Si–C, and Si–O–S bonding due to oxidation of 2D SiC and higher surface area of SiC.

The XRD studies of the bulk and 2D silicon carbide presented in Figure 6a,b show the crystal changes during exfoliation. The XRD pattern of the bulk SiC (Figure 6a) shows peaks at 34.01°, 35.7°, 38.1°, 41.2°, 59.8°, and 65.6°, corresponding to the 6H–SiC planes of (101), (102), (103), (104), (110), and (109) according to the JCPDS card number 00-029-1128. The XRD pattern of 2D SiC (Figure 6b) is slightly similar to that of the bulk SiC, although the main change was due to the broadening and intensity attenuation of the same peaks. The microstructural parameters calculated from the XRD patterns further allow understanding the differences before and after exfoliation: The crystallite size (τ) was reduced in the 2D sample (69.84 nm; σ = ±61.38 nm) compared with the bulk precursor (94.29 nm; σ = ±48.23 nm), which followed the material’s size reduction and the fragmentation of the coherent domains. Conversely, the dislocation density (δ) drastically increased from the bulk (0.2516 × 10^14^ m^2^; σ = ±0.3095 nm) to exfoliated (26.2407 × 10^14^ m^2^; σ = ±103.0888) material, reflecting the highly defective crystal lattice due to the thin-sheet nature of the obtained particles, where edge effects were predominant. Moreover, the microstrain (ε) in the bulk SiC had a value of ε = 1.3703 × 10^−3^ (σ = ±1.0174), indicating a relaxed crystal lattice. However, the lattice structure in the 2D SiC (ε = 6.2103 × 10^−3^; σ = ±18.0526) was stressed due to local defects. The standard deviations in the 2D material were higher than those in the bulk material, which further supports the heterogeneity in the sheet size of the exfoliated SiC and the lattice deformation due to shear forces from the US probe.

As mentioned by Chabi et al. [22], if the obtained nanoparticles only present a peak at 35.7°, they indicate the presence of monolayer SiC. However, in this work, the broadening and attenuation suggested that the number of layers was reduced; therefore, the expected pattern was similar to that of the bulk material, although with differing crystallinity. FTIR results further clarify the structural changes in the SiC after exfoliation. Figure 6c shows the bulk SiC FTIR spectrum. The peaks at 1680 cm^−1^ and 1560 cm^−1^, related to C=C, and 2340 cm^−1^, related to C=O bonding, are ascribable to the bonding between the C atoms in the lattice. Meanwhile, the favorable surface adsorption of O characteristics of the SiC material confirmed the presence of O in the particles, as seen in the EDX analysis. The peaks at 1056 cm^−1^, 812 cm^−1^, and 485 cm^−1^, corresponding to Si–O, Si–C, and Si–O–Si bonding, respectively, are key peaks of 6H–SiC materials, thus confirming the composition and structure of the bulk precursor. The transition from bulk to nanostructured materials results in changes in the FTIR spectra [37,38], since shifts in peak position, intensity, and broadening, among others, can be detected by the increased surface [39], quantum confinement [40], and changes in the surface chemistry [41]. The two-dimensional SiC FTIR spectra can be seen in Figure 6d, showing the disappearance of the 1680 cm^−1^ peak, which may be due to the breaking of the C=C bond. The increased intensity of the 812 cm^−1^ peak highlights the more stable bonding between Si and C, rather than C=C, which can be attributed to the exfoliation. Another noticeable peak is the 2910 cm^−1^ bond that is attributable to the C–H bonding and demonstrates the preferential adsorption of hydrogen onto the surface of 2D SiC, making it attractive for H storage and production [33,35].

The characterizations presented in our work provide an alternative, energetically viable route for further mass production compared with other reported methods (see Table 1), which, despite achieving atomic thickness, limited further applications and evaluations due to their limited stability, complexity to characterize from the substrate, and low yield [42,43,44,45]. Moreover, our strategy achieves a balance between thickness control and high yield since approximately 50 mg of 2D SiC could be obtained from an initial dispersion of 500 mg, which provides a more practical pathway. Furthermore, since further applications toward energy conversion are gaining attention, we can achieve a viable energy cycle.

## 4. Conclusions

In summary, this report presents an optimized route for the ultrasonic exfoliation of bulk 6H–SiC to obtain multi-layered 2D SiC in no more than 2 h. The TEM and AFM micrographs demonstrated that the synthesized nanosheets were composed of ~20 layers of SiC (with an interlayer spacing of ~0.25 nm), with high purity and crystallinity, as shown by the XRD and EDS diffractograms. The FTIR and Raman spectra demonstrated the changes that occurred in the exfoliation via the structural transformation and, thus, the modification of the chemical bonding. This 2D SiC has no polytypes and presents a direct bandgap due to the electron transfer between the valence electrons of Si to the C atoms. Thus, 2D SiC can be used in a wide variety of energy applications, such as acting as a support for transition metals and overcoming the drawbacks of zero-bandgap 2D materials, such as graphene. Moreover, it can enhance hydrogen storage and energy conversion by giving more active sites and stabilizing other nanomaterials, since experimental work has demonstrated the reversible hydrogenation in SiC nanoflakes and the capacity retention improvement with silicon@carron interlayered structures of up to 1145 mAh·g^−1^ with current densities of 1 C at 500 cycles [46,47,48,49]. These promising results can fulfil the gap in the development of high-energy-density rechargeable energy storage devices to improve the performance of green energy sources.

## Figures and Tables

**Table 1:** Comparison of different synthesis methods for fabrication of 2D SiC with respect to our results.

Method	Precursor and Solvent	Total Synthesis Time	Cleaning	Size (nm)	Height (nm)	Ref.
Liquid exfoliation	6H–SiC powder/DMF	16 h	1000 rpm; 30 min	200	3.5	[42]
Carbothermal	3D graphene foam/SiO	N/A	Not reported	2000	3	[43]
Liquid exfoliation	a-SiC/NMP	24 h	9000 rpm; 30 min	10	2	[44]
Liquid exfoliation	6H–SiC powder/DMF	24 h	1000 rpm; 5 min	200–2000	0.28	[22]
Epitaxial growth	4H–SiC substrate/TaC film	10 min	N/A	N/A	0.28	[45]
Liquid exfoliation	6H–SiC powder/IPA	4 h	13,000 rpm; 30 min	2000	5 nm	This work

## Data Availability

The original contributions presented in this study are included in this article/Supplementary Materials. Further inquiries can be directed to the corresponding authors.

## Supplementary Materials

The following supporting information can be downloaded at https://www.mdpi.com/article/10.3390/nano15191480/s1. Figure S1: Three-dimensional AFM surface view of SiC particles after 30 min of US-w exfoliation; Figure S2: Three-dimensional AFM surface view of SiC particles after 1 h of US-w exfoliation; Figure S3: Three-dimensional AFM surface view of SiC particles after 2 h of US-w exfoliation.
