# Supplementary material for: Optimized Sonochemical Exfoliation of Bulk 6H-SiC for the Synthesis of Multi-Layered SiC Nanosheets
Source: Nanomaterials (Basel). 2025 Sep 27;15(19):1480. doi: 10.3390/nano15191480 (PMC12525759; doi:10.3390/nano15191480)
Supplement: Supplementary file 1 [file nanomaterials-15-01480-s001.zip › nanomaterials-3872463-supplementary.pdf]

Supporting Information

Optimized sonochemical exfoliation of bulk 6H-SiC for the synthesis of multi-layered SiC nanosheets

Eric Fernando Vázquez-Vázquez [1], Yazmín Mariela Hernández-Rodríguez [2], Omar Solorza-Feria [3,*] and Oscar Eduardo Cigarroa-Mayorga [2,*]

[1] Department of Nanoscience and Nanotechnology, CINVESTAV-Instituto Politécnico Nacional, Av. Instituto Politécnico Nacional 2508, Mexico City 07360, Mexico; fernando.vazquezv@cinvestav.mx

[2] Advanced Technologies Department, UPIITA-Instituto Politécnico Nacional, Av. Instituto Politécnico Nacional 2580, Col. Ticomán, Mexico City 07340, Mexico; yazmin.hernandez@cinvestav.mx

[3] Department of Chemistry, CINVESTAV-Instituto Politécnico Nacional, Av. Instituto Politécnico Nacional 2508, Mexico City 07360, Mexico

* Correspondence: osolorza@cinvestav.mx (O.S.-F.); ocigarroam@ipn.mx (O.E.C.-M.)

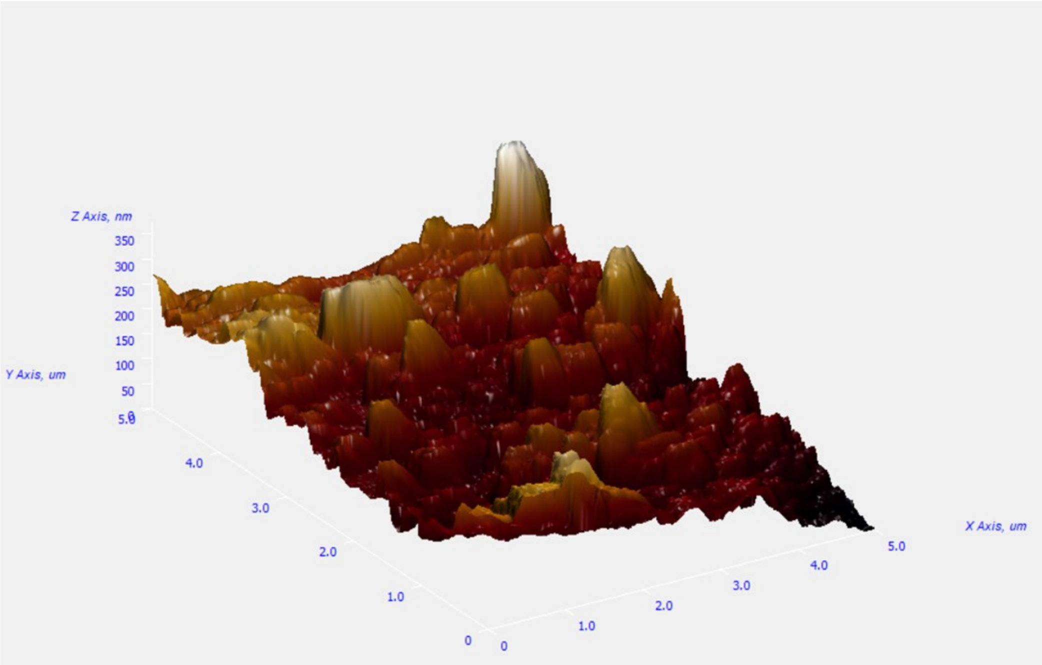

Figure S1. Three-dimensional AFM surface view of SiC particles after 30 min of US-w exfoliation. The image reveals a heterogeneous morphology with large agglomerates and height variations up to ~350 nm. The rough and irregular topography indicates that at this stage the exfoliation process is not yet sufficient to significantly modify the bulk-like structure, consistent with the presence of micrometric domains observed in the corresponding 2D AFM and SEM analyses.

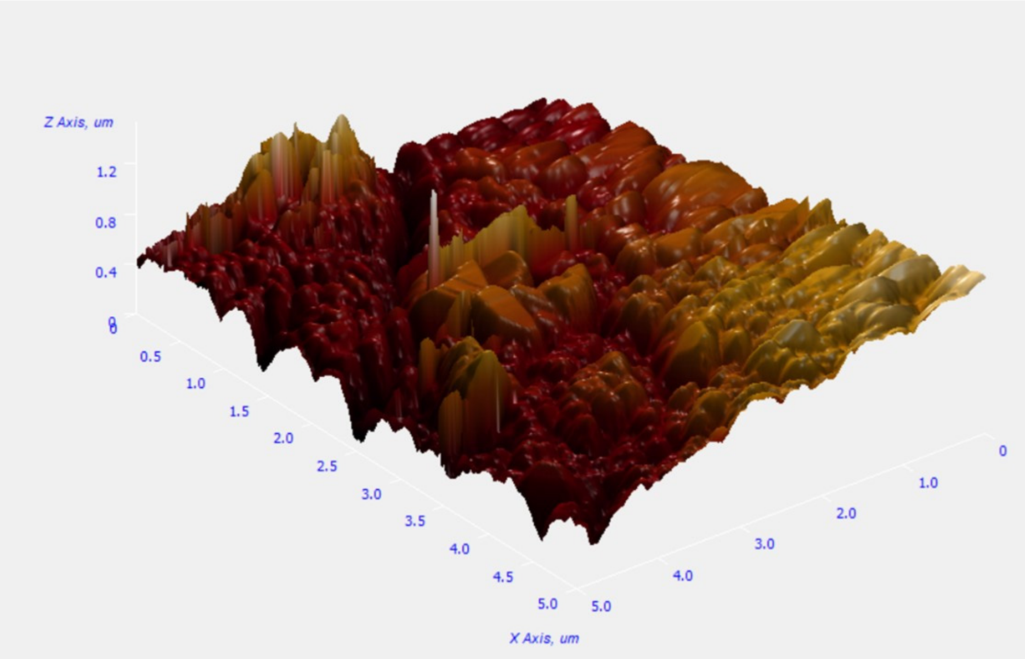

Figure S2. Three-dimensional AFM surface view of SiC particles after 1 h of US-w exfoliation. The image shows a more uniform distribution of smaller features compared to the 30 min exfoliation, with lateral dimensions reduced to the submicron scale and maximum heights below ~1.2 µm. The smoother and more homogeneous topography confirms that increased sonication time enhances the exfoliation efficiency, leading to thinner particles and significant modifications in surface morphology relative to the bulk material.

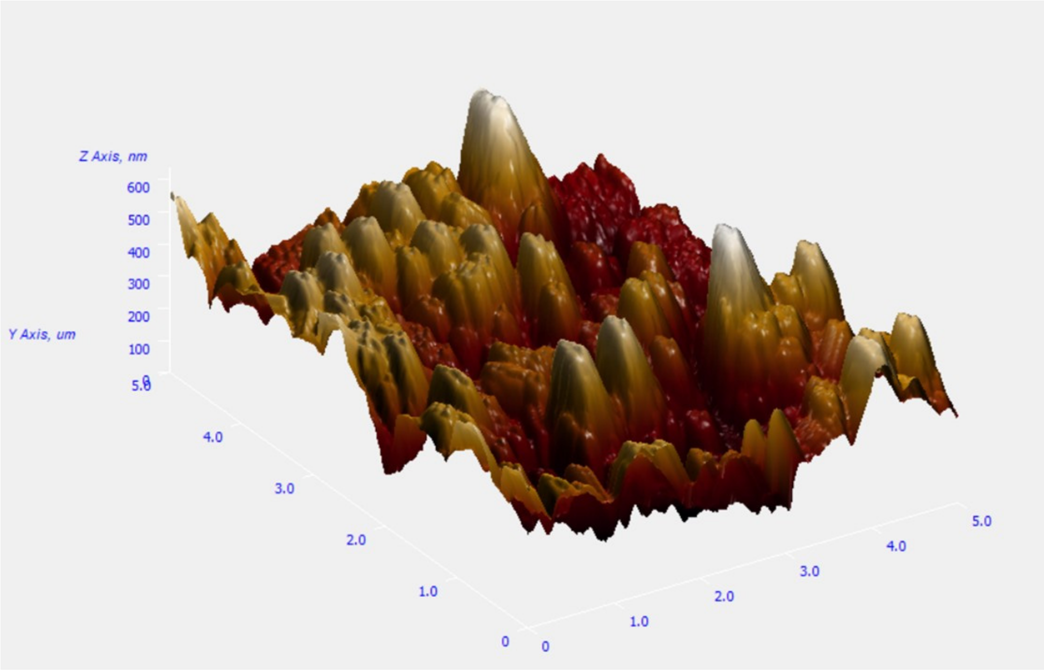

Figure S3. Three-dimensional AFM surface view of SiC particles after 2 h of US-w exfoliation. The image displays well-defined nanosheets with lateral sizes of a few hundred nanometers and reduced heights averaging ~5.5 nm, alongside localized agglomerates reaching ~600 nm. The relatively homogeneous morphology with thinner domains indicates that prolonged sonication promotes the formation of 2D-like SiC structures, although extended times may also generate thicker aggregates at certain regions. These observations are consistent with the evolution toward few-layer SiC sheets as exfoliation time increases.
